# Expression profiling of *AUXIN RESPONSE FACTOR* genes during somatic embryogenesis induction in Arabidopsis 

**DOI:** 10.1007/s00299-017-2114-3

**Published:** 2017-03-02

**Authors:** Barbara Wójcikowska, Małgorzata D. Gaj

**Affiliations:** Department of Genetics, University of Silesia, ul. Jagiellońska 28, 40-032 Katowice, Poland

**Keywords:** ARF5, Auxin signaling, In vitro culture, Monopteros, Organogenesis, Transcription factors

## Abstract

**Key message:**

Extensive modulation of numerous *ARF* transcripts in the embryogenic culture of Arabidopsis indicates a substantial role of auxin signaling in the mechanism of somatic embryogenesis induction.

**Abstract:**

Somatic embryogenesis (SE) is induced by auxin in plants and auxin signaling is considered to play a key role in the molecular mechanism that controls the embryogenic transition of plant somatic cells. Accordingly, the expression of *AUXIN RESPONSE FACTOR* (*ARF*) genes in embryogenic culture of Arabidopsis was analyzed. The study revealed that 14 of the 22 *ARFs* were transcribed during SE in Arabidopsis. RT-qPCR analysis indicated that the expression of six *ARF*s (*ARF5, ARF6, ARF8, ARF10, ARF16*, and *ARF17*) was significantly up-regulated, whereas five other genes (*ARF1, ARF2, ARF3, ARF11*, and *ARF18*) were substantially down-regulated in the SE-induced explants. The activity of *ARF*s during SE was also monitored with GFP reporter lines and the *ARF*s that were expressed in areas of the explants engaged in SE induction were detected. A functional test of *ARF*s transcribed during SE was performed and the embryogenic potential of the *arf* mutants and overexpressor lines was evaluated. *ARF*s with a significantly modulated expression during SE coupled with an impaired embryogenic response of the relevant mutant and/or overexpressor line, including *ARF1, ARF2, ARF3, ARF5, ARF6, ARF8*, and *ARF11* were indicated as possibly being involved in SE induction. The study provides evidence that embryogenic induction strongly depends on *ARFs*, which are key regulators of the auxin signaling. Some clues on the possible functions of the candidate *ARF*s, especially *ARF5*, in the mechanism of embryogenic transition are discussed. The results provide guidelines for further research on the auxin-related functional genomics of SE and the developmental plasticity of somatic cells.

**Electronic supplementary material:**

The online version of this article (doi:10.1007/s00299-017-2114-3) contains supplementary material, which is available to authorized users.

## Introduction

The developmental plasticity of plant somatic cells is widely exploited in green biotechnology in the micropropagation and production of transgenic plants. Among the regeneration pathways that can be induced in a culture of somatic cells/tissue of plants, the process of somatic embryogenesis (SE) is especially attractive for biotechnology purposes as an efficient and fast system for the clonal propagation of many plant species that are of commercial value (Karami et al. 2009). Beside its usefulness in biotechnology, SE was recommended as a model system with which to study the mechanism of embryogenic development in plants (Zimmerman 1993).

The SE process begins with embryonic induction, which is frequently preceded by the de-differentiation of explant tissue (Elhiti et al. 2013). The earliest stage of SE induction attracts the most research attention, because revealing the exo- and endogenous determinants of the embryonic switch contributes to our knowledge of the general mechanism that is involved in developmental cell plasticity and also supports the improvement of the plant regeneration systems that are used in biotechnology.

Among the factors that play a substantial role in SE induction, the exogenous hormone treatment of explant tissue was determined to be required in a culture of most plant species. In over 80% of the protocols on SE induction, auxin was used alone or in combination with other plant growth regulators (Gaj 2004; Karami and Saidi 2010). Thus, an auxin-related molecular mechanism is widely expected to operate during SE induction. As an additional support, a global analysis of SE-transcriptomes indicated that numerous auxin-related genes are transcribed in the embryogenic cultures of different species, including* Picea* sp. (van Zyl et al. 2003; Stasolla et al. 2004), *Zea mays* (Che et al. 2006), *Glycine max* (Thibaud-Nissen et al. 2003), *Solanum tuberosum* (Sharma et al. 2008), and *Arabidopsis thaliana* (Becker et al. 2014).

In auxin-related responses, the genetic components of the auxin-signaling pathway including the AUXIN RESPONSE FACTORs (ARFs) that control the target gene expression in response to auxin were indicated as playing a central role (Teale et al. 2006). *ARF* genes are specific to the plant kingdom and they were identified in the genomes of different plant species, including ferns, gymnosperms, monocots, and dicots (Wang et al. 2012). Twenty-three *ARF* genes were described in Arabidopsis, and except for one pseudogene (*ARF23*), they all encode the TFs that are involved in auxin-mediated responses (Riechmann et al. 2000). The proteins of ARFs contain a DNA-binding domain that is classified as a B3-type and specific to plants, which binds to the TCTCTC motif (AuxRE) found in the promoters of auxin-responsive genes (Guilfoyle et al. 1998). ARFs may activate or repress the target gene expression depending on the amino-acid sequence of the middle region in the functional domain that interacts with DNA. ARFs with domains that are rich in glutamine, serine, and leucine (ARF5, ARF6, ARF7, ARF8, and ARF19) activate transcription while an increased content of serine, proline, leucine, and glycine (ARF1, ARF2, ARF3, ARF4, and ARF9) results in the repression of target transcription (Guilfoyle and Hagen 2001; Tiwari et al. 2001). In addition, ARFs contain the PB1 (Phox and Bem1) domain that is required for the protein–protein interaction with other ARFs and Aux/IAA (Guilfoyle 2015). Recently, a second protein interaction module, the homodimerization DD domain, which is located in DBD, was revealed in at least ARF1 and ARF5 (Boer et al. 2014). The mechanism of auxin-induced gene activation has been well recognized. In the absence of auxin, the Aux/IAA protein interacts with its partner ARF, thereby inactivating any ARF activity; while in the presence of auxin, the Aux/IAA protein is degraded through ubiquitination by the SKP-Cullin-F-box^TIR1/AFB^ (SCF^TIR1/AFBs^) E3 ubiquitin ligase complex, which contains the auxin receptor TRANSPORT INHIBITOR RESPONSE1(TIR1)/AUXIN RECEPTOR F-BOX PROTEINS (AFBs) (Dharmasiri et al. 2005).

The involvement of different ARFs in the regulation of numerous auxin-controlled developmental processes including flowering, leaf senescence, gynoecium and seed formation, root development, vascular tissue formation, and abaxial identity of organs was indicated in Arabidopsis (Guilfoyle and Hagen 2007). An involvement of ARFs in the control of ZE is also evident (Rademacher et al. 2011), including an ARF-dependent suspensor (ARF1, ARF2, ARF6, ARF9, and ARF13) and proper embryo development (ARF1, ARF2, ARF5, ARF6, and ARF7). Consequently, mutations in *ARF* genes were found to strongly disturb zygotic embryo development (Rademacher et al. 2011, 2012).

In contrast to ZE, the role of ARFs in SE remains mostly unknown. In this study, we profiled the expression of all known *ARF* genes during SE that was induced in a culture of Arabidopsis immature zygotic embryo (IZE) explants. Gene expression profiling and analysis of insertional mutants, overexpressor, and reporter lines were used to identify SE-involved *ARF*s. The *ARF*s were found to significantly differ in their activity in the embryogenic culture and the impact on somatic embryo induction. The results confirm that numerous *ARFs* control the embryogenic transition that is induced in somatic cells. Of the *ARFs* that operate during embryogenic transition that is induced in vitro, *ARF5* seems to be of particular importance for SE.

## Materials and methods

### Plant material

The Columbia (Col-0) genotype and the transgenic lines including insertional mutants, GFP reporter and overexpressing lines of *Arabidopsis thaliana* (L.) Heynh., were used (Supplementary Table 1). All seeds, except for 35S::ARF5, which were kindly provided by Dr. Ive de Smet (Department of Plant Systems Biology, VIB, Ghent, Belgium), were supplied by NASC (The Nottingham Arabidopsis Stock Centre).

### Plant growth and in vitro culture conditions

The plants that were used as the source of IZE explants were grown in 42 mm diameter Jiffy-7 peat pots (Jiffy) in a ‘walk-in’ type phytotron under controlled condition at 22 °C under a 16 h photoperiod of 100 µM m^− 2^ s^− 1^ white, fluorescent light. Plant materials that were grown in sterile conditions were kept at 23 °C under a 16 h photoperiod of 40 µM m^− 2^ s^− 1^ white, fluorescent light.

### Somatic embryogenesis

Immature zygotic embryos in the late cotyledonary stage of development were used as explants for the in vitro culture and the standard protocol was applied to induce SE in Arabidopsis (Gaj 2001). Ten IZEs were cultured in a Petri dish (35 mm) on an agar-solidified (8 g L^− l^) induction medium containing basal B5 micro- and macro-elements (Gamborg et al. 1968) and 20 g L^− 1^ sucrose. IZE explants were cultured on an SE-induction auxin medium (E5) with 5 µM 2,4-D (2,4-dichlorophenoxyacetic acid) and on a control, hormone-free (E0) medium. Ten explants were cultured in one Petri dish and thirty explants in three replicates in each culture combination were analyzed. The capacity for SE was evaluated in 3-week-old cultures. Two parameters of embryogenic potential were evaluated: SE efficiency—frequency of the explants that produced somatic embryos and SE productivity—the average number of somatic embryos that developed per embryogenic explant.

### Shoot organogenesis

To induce shoot regeneration via organogenesis (ORG), the IZEs were incubated for 7 days in a liquid callus induction medium (CIM), then the cotyledons were cut off and cultured on solid shoot induction media (SIM-C) according to Kraut et al. (2011). The CIM medium contained a basal composition of a B5 medium (Gamborg et al. 1968), 0.5 g L^− 1^ MES, 20 g L^− 1^ glucose, 2.2 µM of 2,4-D, and 0.2 µM of kinetin (Kraut et al. 2011). The SIM-C medium contained micro-elements of MS (Murashige and Skoog 1962), macro-salts, vitamins of a B5 medium (Gamborg et al. 1968), and was supplemented with 30 g L^− 1^ sucrose, 0.5 µM of NAA (1-naphthaleneacetic acid) and 4.4 µM of BAP (6-benzylaminopurine). The explant capacity for ORG was evaluated in 3-week-old cultures. Two parameters of culture morphogenic potential were evaluated: ORG efficiency—frequency of the explants that produced shoots and ORG productivity—the average number of shoots that developed per explant.

### RNA isolation and gene expression analysis

An RNAqueous Kit (AMBION) was used to isolate total RNA from the IZE explants that were induced on the SE induction (E5), callus induction (CIM), shoot induction (SIM), and the hormone-free (E0) medium on days 0, 3, 5, 10, and 15 of the culture. Depending on the age of the culture, from 250 (0 day) to 4 (15 days), explants were used for the isolation of RNA. The concentration and purity of RNA were evaluated with a ND-1000 spectrophotometer (NanoDrop). To prevent DNA contamination, the RNAs were treated with RQ1 RNase-free DNase I (Promega) following the manufacturer’s instructions. First-strand cDNA was produced in a 20 µL reaction volume using a RevertAid First-Strand cDNA Synthesis Kit (Fermentas).

The product of the reverse transcription was diluted with water in a 1:1 ratio and 1 µL of this solution was used for RT-PCR. To quantify the expression of *ARF* genes, Real-Time quantitative RT-PCR (RT-qPCR) method was employed. The product of the reverse transcription was diluted in 3:1 ratio than 2.5 µL was used for reactions. RT-qPCR was carried out in a 10 µL reaction volume using a LightCycler® 480 SYBR™ Green I Master (Roche) kit, a LightCycler® 480 Multiwell Plate 96, and MultiwellSealin Foil (Roche).

A LightCycler® 480 System (Roche) real-time detection system was used under the following reaction conditions: denaturation one repeat of 5 min at 95 °C, followed by 45 repeats of 10 s at 95 °C, 20 s at 58 °C, and 10 s at 72 °C. Denaturation for melt curve analysis was conducted at 95 °C followed by 5 s, at 65 °C by 1 min, and 98 °C (0.11ºC/s for fluorescence measurement). Cooling was at 40 °C for 10 s.

Relative RNA levels were calculated and normalized to internal controls, the *AT1G07920 ELONGATION FACTOR1α* (*EF1α*) gene encoded GTP-binding elongation factor (Reid et al. 2006) and *AT4G27090* (*TIN*) gene encoded 60 S ribosomal protein (Thellin et al. 1999). Fold change values were calculated using the comparative 2^− ΔΔCt^ method. The control gene exhibited a constant expression pattern with Cp = 24 ± 1 in all of the tissue samples that were analyzed. The primers (Supplementary Table 1) used for expression profiling of the genes were designed using the QuantPrime tool (http://www.quantprime.de/). The plant tissues for gene expression analysis were produced in three biological replicates and two technical replicates of each repetition were carried out. All of the information about the RNA and RT-qPCR quality and methodology are presented in Supplementary Table S1.

### Microscopy

Analysis of GFP signal was carried out using a Nikon Eclipse Ni-E/Ni-U fluorescent microscope system. GFP fluorescence was excited using halogen lamphouses with a 100–240 VAC (Prior Lumen200) and a wavelength of 488 nm. Photographic documentation was created from images that were recorded with a Nikon Digital Sight DS-Fi2 with DS-U3 camera. Image processing was performed using the NIS-Elements F computer program version 4.0.

### Statistical analysis

The student t test was used to calculate any significant differences (at *P* = 0.05) between the combinations that were being compared.

## Results

### Design of the experiment

**Fig. 1:**
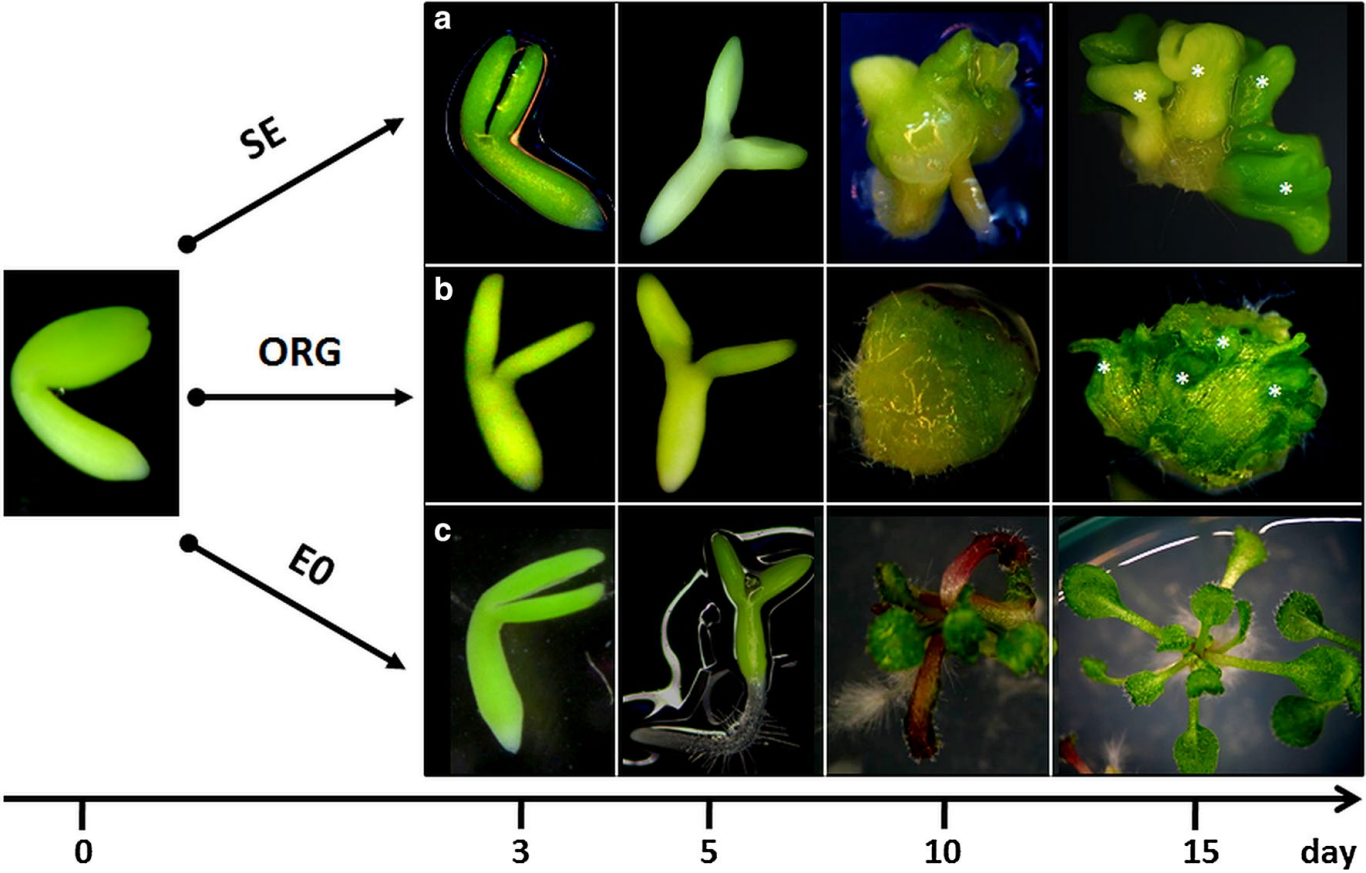
Diverse morphogenic pathways that were induced in vitro in a culture of Arabidopsis IZE explants. IZE explants of Col-0 that were cultured on different media resulted in: somatic embryo development, SE (**a**), shoot regeneration, ORG (**b**), and seedling development, E0 (**c**); the tissue for the analysis of *ARFs* expression was sampled at different time points of the cultures, including 0, 3, 5, 10, and 15 days. *Asterisk* regenerated somatic embryos or shoots

A culture of IZEs results in SE, shoot organogenesis (ORG), or seedling development in Arabidopsis, and the type of morphogenic response of explants mainly depends on the hormonal content of the media that is used (Kraut et al. 2011). To identify the *ARF*s that are differently expressed during SE, explant tissues that were induced on various media towards SE, ORG, and seedling development were sampled (Fig. 1). The analysis covered the different stages of the culture, including freshly isolated (0 day) and media-cultured (3–15 days) IZE explants. In the SE culture, samples taken on days 3 and 5 of the culture are related to the SE-induction stage, while the later culture stages, which are related to the formation and development of somatic embryos, correspond to days 10 and 15 of the culture, respectively.

### Expression profiling of the *ARFs* transcribed in the embryogenic culture

The analysis of the *ARFs* expression based on qualitative RT-PCR reaction, which was monitored at different stages of SE, indicated that the majority (14) of the 22 analyzed genes were transcribed in the IZE explants and the embryogenic cultures that were derived, including *ARF1, ARF2, ARF3, ARF5, ARF6, ARF7, ARF8, ARF9, ARF10, ARF11, ARF16, ARF17, ARF18*, and *ARF19* (Fig. S1). The RT-qPCR analysis revealed that the *ARFs* that were transcribed in the embryogenic culture displayed diverse patterns of expression and the genes differed distinctly in the levels and profiles of their transcription (Fig. S2). *ARF5* and *ARF10* were found to have the highest transcript accumulation in the SE culture, while in contrast, the activity of *ARF7* and *ARF11* was almost 7–9 threshold cycles lower. Most of the SE-transcribed *ARFs* displayed a substantial modulation of their expression level during SE in relation to 0 day and genes that displayed both increased and decreased transcription levels in specific SE-stages were indicated.

### *ARFs* up-regulated during SE

**Fig. 2:**
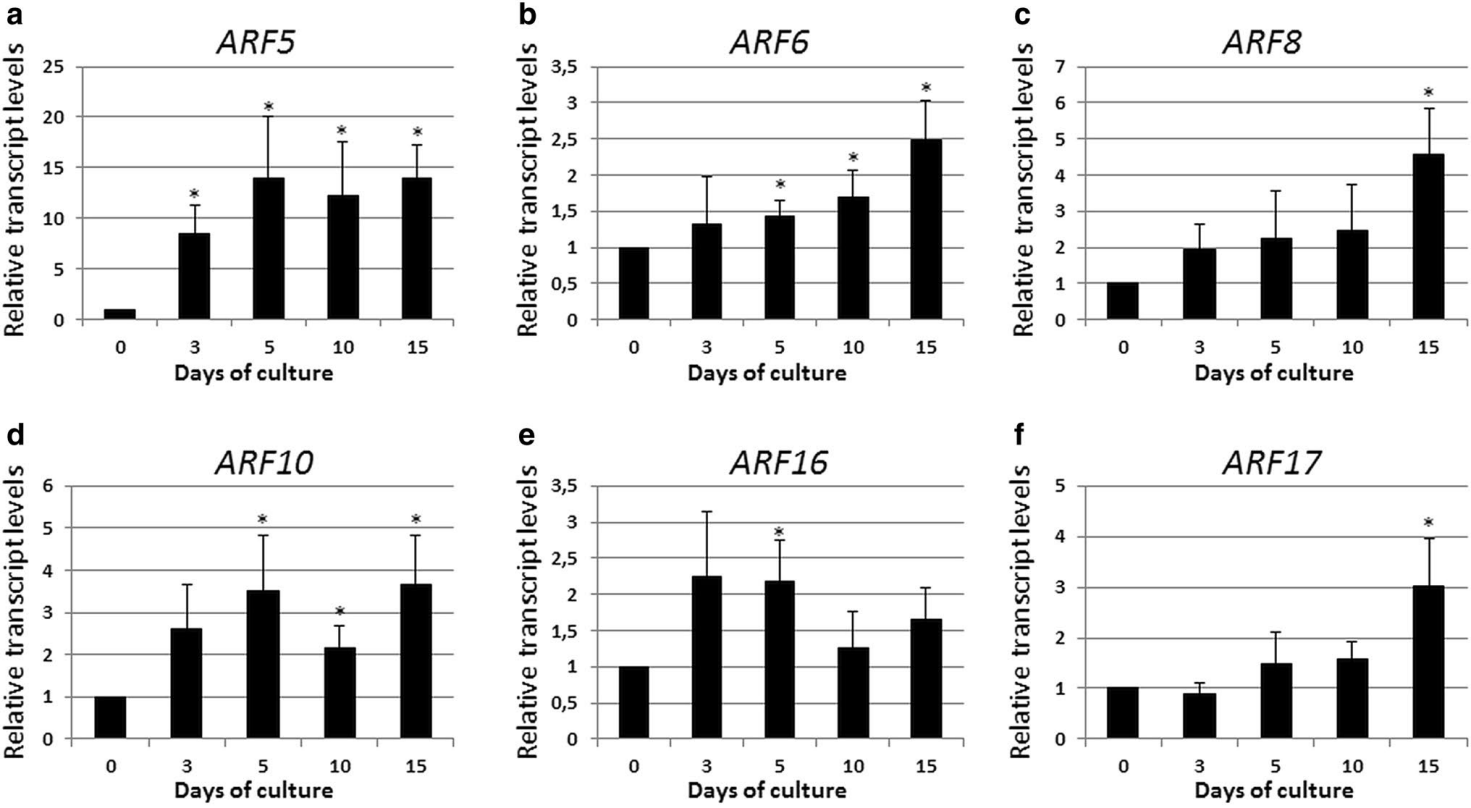
*ARF5* (**a**), *ARF6* (**b**), *ARF8* (**c**), *ARF10* (**d**) *ARF16* (**e**), and *ARF17* (**f**) of up-regulated expression during the SE process. IZE explants of Col-0 were cultured on an E5 medium and the tissue was sampled on the 0, 3, 5, 10, and 15 days of the culture. The relative transcript level was normalized to the internal control (*TIN* gene) and calibrated to the 0 day culture. *Asterisk* expression level significantly different to that observed at 0 day at *P* < 0.05. Means and SD for three biological replicates are shown

Six of the *ARF*s were found to be significantly up-regulated at different (3–15 days) time points of the embryogenic culture and the genes with a stimulated transcription in the early (3–5 days) or late (10–15 days) stages of SE could be distinguished (Fig. 2). The majority (four) of the SE-stimulated *ARF*s including *ARF5, ARF6, ARF10*, and *ARF16* displayed a distinct up-regulation during the inductive stage of SE (3–5 days), while two other genes, *ARF8* and *ARF17*, showed a significantly increased level of expression in the advanced stages of SE that were associated with the formation and development of the somatic embryo (10–15 days).

Among the *ARF*s with an SE-stimulated expression, the *ARF5* gene was found to be especially highly activated and its transcript level showed a distinguishably sharp increase in the early SE (up to 14-fold). This observation suggested that *ARF5* strongly affects the embryogenic transition in explant cells.

### *ARFs* down-regulated during SE

**Fig. 3:**
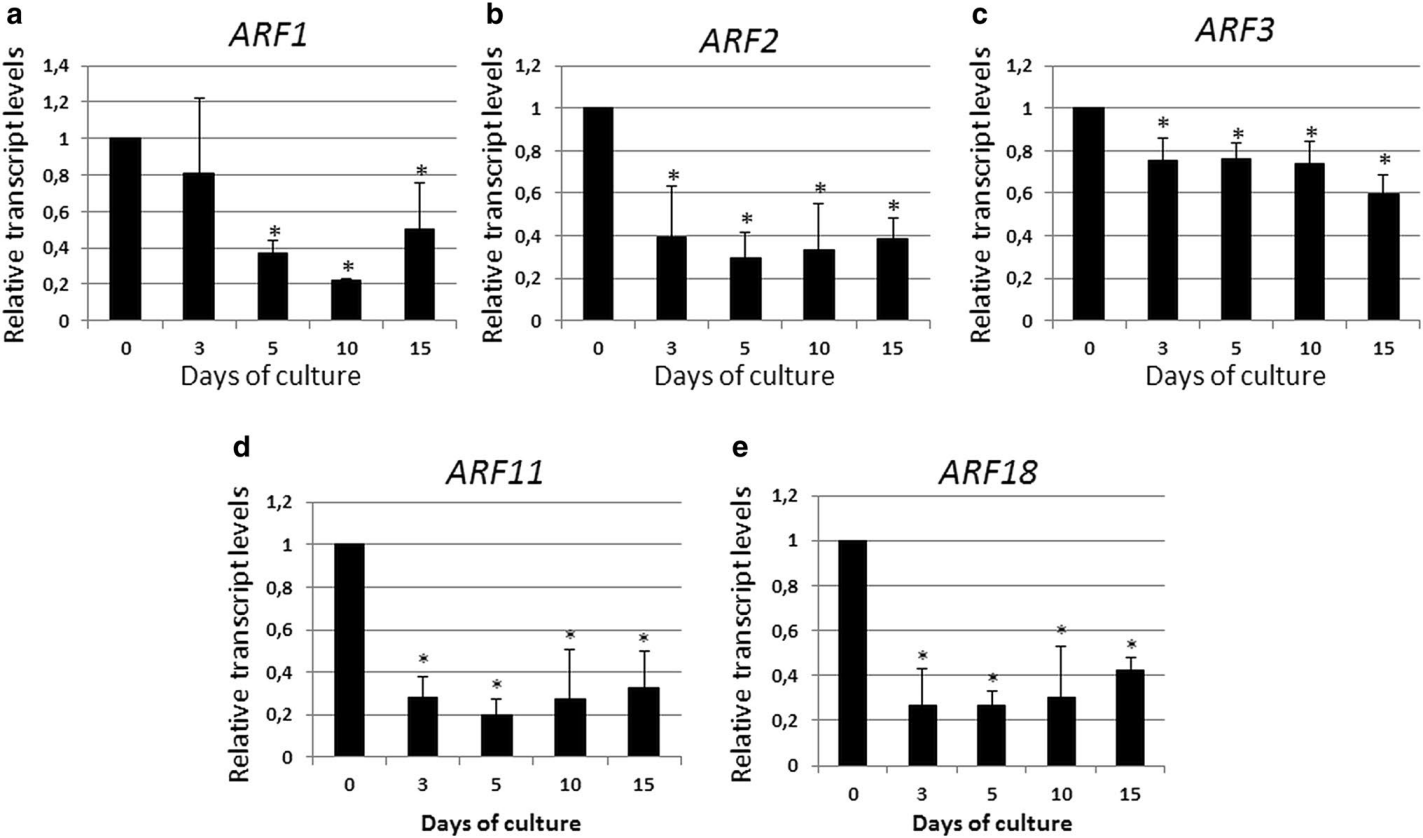
*ARF1* (**a**), *ARF2* (**b**), *ARF3* (**c**), *ARF11* (**d**), and *ARF18* (**e**) of down-regulated expression during the SE process. IZE explants of Col-0 were cultured on an E5 medium and the tissue was sampled on the 0, 3, 5, 10, and 15 days of the culture. The relative transcript level was normalized to the internal control (*TIN* gene) and calibrated to the 0 day culture. *Asterisk* expression level significantly different to that observed at 0 day at *P* < 0.05. Means and SD for three biological replicates are shown

Five of the *ARF* genes including *ARF1, ARF2, ARF3, ARF11*, and *ARF18* were observed to display a significant reduction in their activity in the early stage of SE induction (3–5 days), which also remained low in the advanced culture (10–15 days) (Fig. 3). Among these genes, the activity of *ARF11* was found to be the most reduced in the embryogenic culture and transcripts of this gene were up to four-times less abundant in the SE-induced explants than in the freshly isolated explants.

### *ARFs* with a stable expression level during SE

The observed during SE changes in transcript level were found not to be significant for three genes: *ARF7, ARF9*, and *ARF19*, and thus, these *ARF*s were considered to have a stable expression during SE (Fig. S3).

### Auxin treatment and *ARF*s expression

**Fig. 4:**
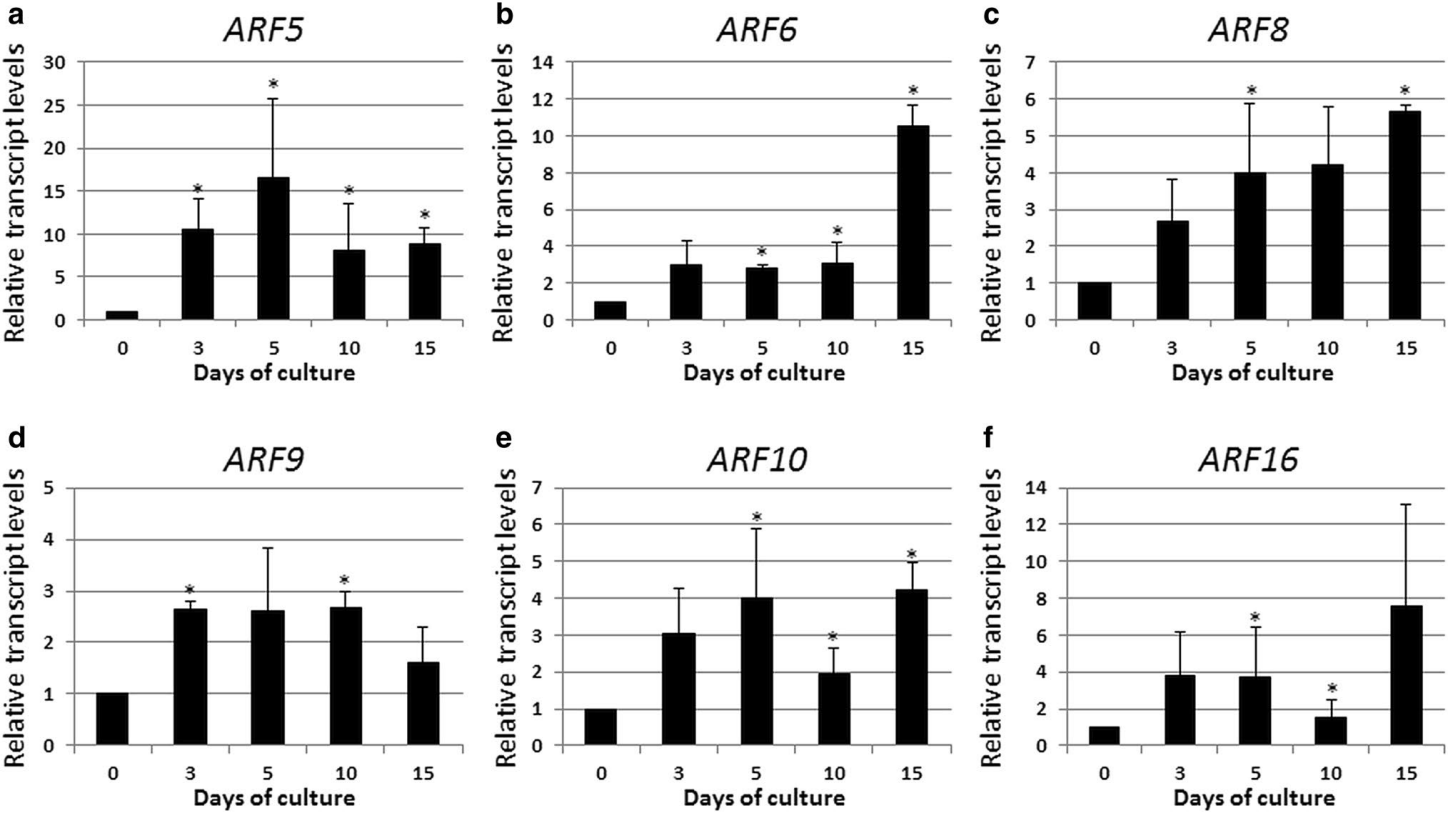
*ARF5* (**a**), *ARF6* (**b**), *ARF8* (**c**), *ARF9* (**d**), *ARF10* (**e**), and *ARF16* (**f**) genes of auxin-stimulated expression during the SE process. IZE explants of Col-0 were cultured on an E5 medium and the tissue was sampled on the 0, 3, 5, 10, and 15 days of the culture. Relative transcript level was normalized to the internal control (*TIN* gene) and calibrated to the gene expression in explants that had been induced on an auxin-free (E0) medium. *Asterisk* expression level significantly different to that observed on the E0 medium at *P* < 0.05. Means and SD for three biological replicates are shown

To reveal the *ARF*s with an auxin-regulated expression, in addition to the E5 auxin medium with a SE-promoting effect, the gene expression was also evaluated in IZEs that had been cultured on the auxin-free medium (E0) that resulted in seedling development. A comparison of the *ARF* expression profiles on the E5 versus E0 medium indicated that six *ARF* genes (*ARF5, ARF6, ARF8, ARF9, ARF10*, and *ARF16*) had a distinctly higher expression level under auxin treatment (Fig. 4). The majority of these genes (*ARF5, ARF6, ARF8, ARF10*, and *ARF16*) also showed a significantly increased transcript abundance in the embryogenic culture (Fig. 3). Thus, auxin treatment appears to be responsible for the distinct up-regulation of most of the *ARF* genes that are observed during SE. Among the *ARF*s that are auxin-stimulated and up-regulated during SE, *ARF5* was ascertained to be the most highly auxin-responsive and its expression was up to 16-times higher (5 days) on E5 than on the E0 medium. *ARF9* was also identified among auxin-stimulated *ARFs;* however, its activity was not significantly increased between 0–15 days of SE culture. By contrast, *ARF17*, which was observed to be up-regulated during SE (Fig. 2), was found not to be stimulated in response to auxin treatment, which implies that other factors besides auxin may be responsible for the activation of *ARF* genes during SE.

### *ARFs* differentially expressed in SE versus ORG

**Fig. 5:**
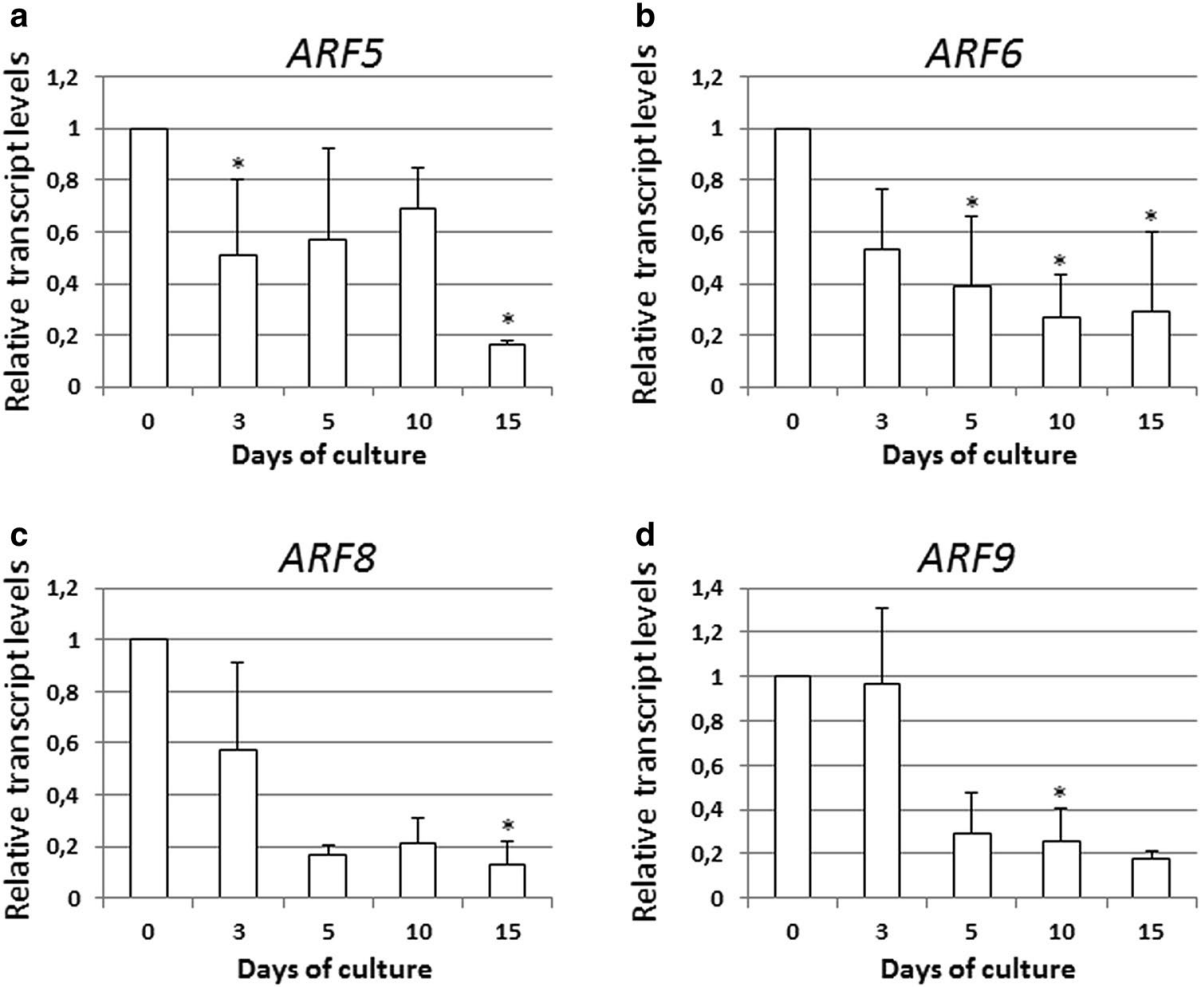
*ARF* genes with a distinctly lower expression level in ORG than in the SE culture: *ARF5* (**a**), *ARF6* (**b**), *ARF8* (**c**), and *ARF9* (**d**). Relative transcript level was normalized to the internal control (*TIN* gene) and calibrated to the *ARFs* expression during the SE process. *Asterisk* expression level significantly different to that observed in the SE culture at *P* < 0.05. Means and SD for three biological replicates are shown

To determine whether the patterns of *ARFs* expression that were observed in the embryogenic culture are unique for SE induction or whether they result from the general processes of de- and re-differentiation that are expected in hormone-treated explant tissue, IZE explants that had undergone ORG were also studied. The results revealed that all 14 *ARF*s that were transcribed in the embryogenic culture were also expressed in the ORG culture (Fig. S4). Comparison of the gene expression levels during SE and ORG, using the RT-qPCR method, resulted in the identification of *ARF* genes that displayed distinctly different levels of activity in these processes. Four of the *ARF*s (*ARF5, ARF6, ARF8*, and *ARF9*) were observed to show a lower expression level during ORG than in the SE culture **(**Fig. 5). Three of them (*ARF5, ARF6*, and *ARF8*) appear to be especially interesting in terms of their possible involvement in SE due to their considerably higher expression in the embryogenic culture.

### Summary of the results of the PCR/qPCR analysis

Comparison of the *ARF* expression profiles in the explants that were subjected to different culture conditions that promote SE and ORG showed that similar *ARF*s were transcribed in these processes and that the set of the SE/ORG-expressed genes included a majority (14 of 22) of the analyzed genes (Supplementary Table S2). The expression patterns of five of these *ARF*s (*ARF6, ARF8, ARF9, ARF16*, and *ARF19*) were distinctly different in SE compared to ORG and *ARF5, ARF6, ARF8*, and *ARF9* displayed a significantly higher transcript accumulation in SE than in ORG. Except for three genes (*ARF7, ARF9*, and *ARF19*) that were transcribed at a stable level in the embryogenic culture, a predominant number of *ARF*s (11/14; 79%) displayed a significantly modulated expression profile during SE and a similar number of the up- (6) and down- (5) regulated *ARF*s were observed. In line with the key role of auxin in the SE induction mechanism, it was found that auxin treatment might have been the cause of the increased expression of almost all of the genes that were up-regulated during SE with the exception of *ARF17*.

### GFP reporter analysis of *ARF *expression during SE-induced explants

The spatio-temporal pattern of *ARFs* expression in the IZE explants undergoing SE induction was analyzed using the GFP reporter lines. In particular, the explant areas that are involved in SE induction, i.e., the cotyledons and the vicinity of SAM (Kurczyńska et al. 2007) were inspected in terms of the GFP signal.

**Fig. 6:**
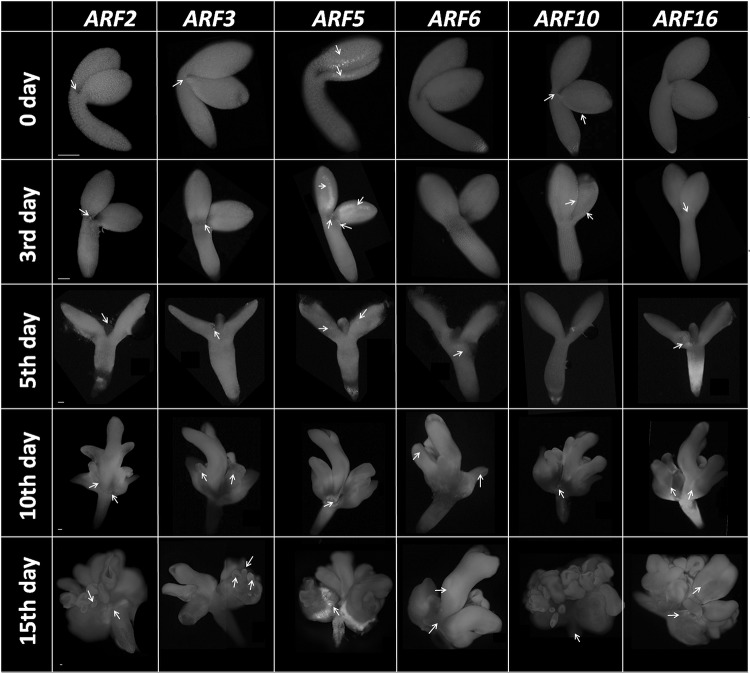
GFP-monitored *ARF2, ARF3, ARF5, ARF6, ARF10*, and *ARF16* expression in the IZE explants that had been cultured for 0, 3, 5, 10, and 15 days on an SE-induction medium. The GFP signal in the SE-involved parts of the explants is indicated by an *arrow*

The GFP signal was identified in nine reporter lines that monitored expression of *ARF1, ARF2, ARF3, ARF5, ARF6, ARF10, ARF16, ARF18*, and *ARF19*, and in six of them, a reporter signal was detected in the IZE regions that are involved in SE induction (Fig. 6). In freshly isolated IZE explants (0 day), a GFP signal was detected for *ARF2, ARF3, ARF5*, and *ARF10*. During the early SE induction (3–5 days), *ARF5, ARF6, ARF10*, and *ARF16* were observed to be expressed along the cotyledons and in the vicinity of SAM and an especially strong GFP signal was seen in the *ARF5* reporter line. SE-related expression in the early culture (3–5 days) also displayed two others genes, *ARF2* and *ARF3*, with the GFP signal localized exclusively in the vicinity of SAM. In the advanced embryogenic culture (10–15 days), a weak GFP signal was seen in the developing somatic embryos in some of the reporter lines (*ARF2, ARF3, ARF6*, and *ARF16*), while a strong fluorescence indicative of the expression of *ARF2, ARF5, ARF10*, and *ARF16*, was found to be associated with the callus tissue produced at the base of the somatic embryos and in the explant parts not engaged in SE (hypocotyl and root).

## Functional test of SE-transcribed *ARFs*

### *arf *mutants

**Fig. 7:**
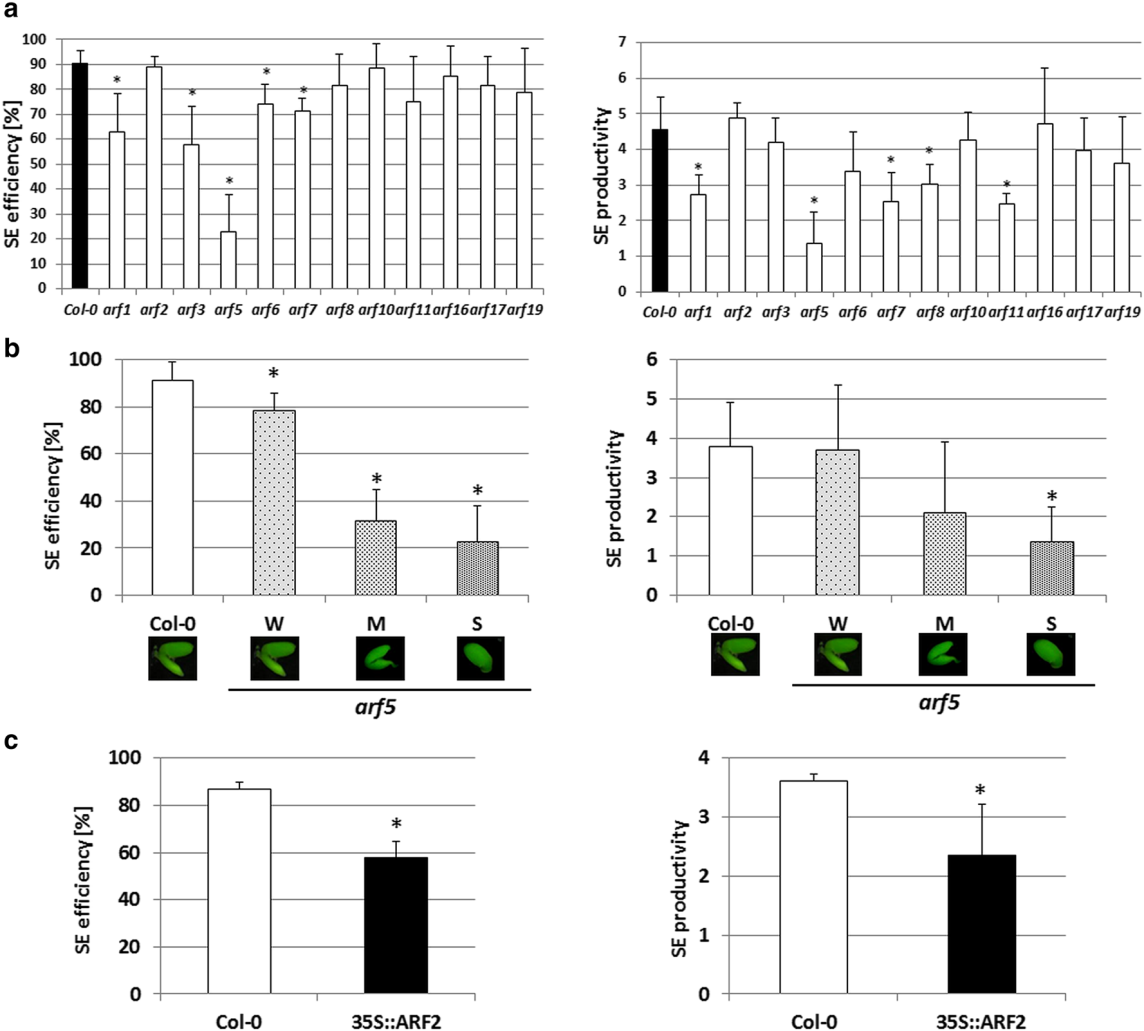
Functional test of SE-transcribed *ARFs*. The embryogenic potential, which was measured by SE efficiency and SE productivity, was evaluated in cultures derived from: *arf* insertional mutants (**a**); *arf5* explants of a weak (W), middle (M), and strong (S) mutant phenotype (**b**); 35S::ARF2 overexpressor line (**c**). *Asterisk* embryogenic capacity significantly different to that observed in the Col-0 control culture at *P* < 0.05. Means and SD for three biological replicates are shown

To further explore the involvement of *ARF* genes in SE, the *arf* insertional mutants were analyzed in terms of their capacity for SE induction. In total, 12 of the *arf* mutants (*arf1, arf2, arf3, arf5, arf6, arf7, arf8, arf10, arf11, arf16, arf17*, and *arf19*) were evaluated and the analysis indicated that most (seven) of the *arf* mutants were impaired in their embryogenic response (Fig. 7a). Three of the mutants, *arf1, arf5*, and *arf7*, displayed a significant reduction in both of the parameters of SE capacity, i.e., SE efficiency and SE productivity. The other mutants were defective in SE efficiency or SE productivity, and accordingly, *arf3* and *arf6* displayed a reduced frequency of SE-responsive explants, while *arf8* and *arf11* were observed to produce a significantly fewer number of somatic embryos per responsive explant.

The *arf5* mutant was found to be the most hampered in its embryogenic response and the severity of developmental defects that were displayed by the IZEs of the mutant (classified as a weak, medium, and strong mutant phenotype) was positively correlated with a reduction of the SE response (Fig. 7b). An extreme decrease in embryogenic capacity (over 80%) was observed in the culture of IZEs that had a strong, the so-called *monopteros*, mutant phenotype, which is characterised by a single cotyledon and the lack of the root meristem and hypocotyl (Odat et al. 2014).

### ARF overexpressors

Further evidence of the considerable role of *ARF5* in SE induction was provided by the analysis of the 35S::ARF5 line. Overexpression of *ARF5* results in the strongly defective development of plants including distorted inflorescences, which may terminate in pin-shaped inflorescence tips and high plant sterility (Hardtke et al. 2004). Therefore, only 20 IZEs were collected from 12 plants and cultured on the SE-induction medium. None of the explant overexpressing *ARF5* was capable of developing somatic embryos and instead only a callus was formed.

In addition to a highly expressed *ARF5* gene, the effect of overexpression on an explant’s capacity for SE was also evaluated in the 35S::ARF2 line that overexpressed an ARF that had a substantially decreased transcription during SE. The analysis indicated that the increased activity of *ARF2* resulted in a substantial reduction in embryogenic response and both SE efficiency and productivity were significantly reduced (Fig. 7c).

### Candidate targets of ARFs in SE

We assumed that the targets of SE-modulated *ARF*s might be identified within the auxin-responsive *TF* genes that play a regulatory role in SE. Accordingly, using the Arabidopsis *cis*-regulatory element database (AGRIS; http://arabidopsis.med.ohio-state.edu/AtcisDB/), we found that an Auxin Response Element (AuxRE) is present in the promoters of the majority (60%) of SE-modulated *TF*s (Gliwicka et al. 2013) including the genes that were indicated to have an essential function in embryogenic transition such as *LEAFY COTYLEDON* (*LEC1* and *LEC2*; Gaj et al. 2005), *FUSCA3* (*FUS3*; Ledwoń and Gaj 2011), *WUSCHEL* (*WUS*; Zuo et al. 2002), *BABY BOOM* (*BBM*; Boutilier et al. 2002), and *MYB115* (Wang et al. 2009). It remains to be revealed which of the candidate ARFs bind to the promoters of the key regulators of SE. Among the ARF-*TF* regulatory interactions that operate during SE, the direct regulation of *LEC2* by ARF5/MP cannot be excluded due to the significantly reduced *LEC2* transcript level that was found in an *mp*/*arf5* mutant that had a seriously defective embryogenic response (Fig. S5).

To gain more insight into the ARF-mediated regulatory interactions that operate in SE, we also verified the possibility of an auto-feedback regulation of *ARFs* (Lau et al. 2011; Okushima et al. 2005). Accordingly, we used the AGRIS analytical tool to search for AuxREs in the promoters of the *ARF*s that were found to display a significantly and SE-modulated expression level in this study. The analysis showed the presence of AuxREs in the majority (85%) of the SE-modulated *ARF*s (Supplementary Table 3). Thus, the complexity of the ARF-controlled regulatory relations that operate in SE might be enhanced by the auto-feedback regulated transcription of the candidate *ARF*s.

## Discussion

### Numerous *ARFs* contribute to SE-transcriptome via distinctly different expression patterns

Auxin signaling is believed to play a pivotal role in plant development including morphogenic processes that are induced in in vitro cultured explants (reviewed in Ikeuchi et al. 2016; Weijers and Wagner 2016). Consistent with this belief, the global analysis of the SE-transcriptome of Arabidopsis indicated that components of the auxin signaling pathway, including *AUX*/*IAA* and *ARF*, which are the main regulators of the auxin signaling, together with auxin-responsive target genes are extensively modulated during SE induction (Gliwicka et al. 2013; Wickramasuriya and Dunwell 2015). This study, whose aim was to identify the candidate ARFs that are involved in SE induction indicated that most (14/23) of the *ARF*s encoded in Arabidopsis genome were transcribed in the embryogenic culture and that the great majority of these (11) displayed a significantly and SE-modulated expression level.

ARF proteins are believed to activate or repress the target genes (Weijers and Wagner 2016) and in the transient and heterologous assays, individual ARFs are classified as activators/repressors of the gene transcription (Tiwari et al. 2001). According to this classification, the *ARF*s of SE-modulated expressions that were identified in this study represent both the activators (*ARF5, ARF6, ARF7, ARF8*, and *ARF19*) and repressors (*ARF1, ARF2, ARF3*, and *ARF9*) of the target gene transcription. However, it is necessary to experimentally validate the regulatory relationship between the ARFs and their SE-targeted genes among which the auxin-responsive genes encoding the TFs that have a documented function in SE such as LEC2, FUS3, WUS, BBM, and MYB115 (reviewed in Nowak and Gaj 2016) might be considered.

It is also worth noting that more complex behaviours of ARFs in SE than simply the direct activation/repression of the target genes may exist including binding ARFs with repressor and activator functions to the same AuxREs in the same target gene (Vernoux et al. 2011; Bargmann and Estelle 2014; Chandler 2016). Consistent with this expectation, we observed a distinct up-regulation of several AuxRE-containing *ARF*s (*ARF5, ARF6, ARF8, ARF10*, and *ARF16*) in response to auxin treatment. In further support for a vital role of auxin treatment in the regulation of *ARFs* expression during SE, we observed that *ARF*s that lacked AuxRE were not transcribed (*ARF14* and *ARF22*) or that their expression was not modulated (*ARF7*) in the embryogenic culture of Arabidopsis.

In addition to auxin, various abiotic stresses were also indicated as modulating the transcription of *ARF*s (Tian et al. 2004; Hannah et al. 2005; Jain and Khurana 2009; Blomster et al. 2011; Naser and Shani 2016). Thus, the stress conditions including ROS (Reactive Oxygen Species), which are actively produced under embryogenic culture, may control the expression of *ARF*s during SE induction (Zavattieri et al. 2010; Blomster et al. 2011).

*ARF-*mediated transcriptional responses are believed to be highly specific not only to the developmental process but also to tissue and cell type (Weijers et al. 2005), and this raises a question about the extent of the similarities of the ARF-mediated auxin responses that are activated in different SE systems. A comparison of the transcriptional profiles of *ARF*s that were identified in the embryogenic cultures of various plants infers that the contribution of the individual *ARF*s to the mechanism of SE induction may differ between embryogenic cultures (Thibaud-Nissen et al. 2003; Ooi et al. 2012; Yang et al. 2012; Xu et al. 2013; Lin et al. 2015; Indoliya et al. 2016; Zheng et al. 2016).

Considering the apparent molecular similarities of SE to ZE (Dodeman et al. 1997; Winkelmann 2016), we found the expression patterns of some *ARF*s to be convergent in both the embryogenic culture and in the ZE of Arabidopsis, and, e.g., a significant up-regulation of *ARF5* and *ARF6* in both of these processes is evident (present results *versus* http://www2.bri.nrc.ca/plantembryo). In ZE, these closely related *ARF*s act synergistically; however, a mutation in only in one of these genes, *ARF5*, causes substantial defects in the zygotic embryo (Rademacher et al. 2011). In contrast to ZE, we found the *arf6* mutant to be significantly impaired in its capacity for SE and these inconsistent mutant phenotypes suggest different contributions of ARF6 in the regulation of SE and ZE. In particular, different roles and regulatory relations of individual *ARF*s can be expected in the very early stages of these processes. In contrast to the ZE that originates from a totipotent zygotic cell, SE induction involves the re-programming of already differentiated somatic cell/cells that respond to the auxin treatment. As a result, IAA is accumulated in the SE-induced cells (Michalczuk et al. 1992; Ribnicky et al. 2002; Kurczyńska et al. 2007; Wójcikowska and Gaj 2015) and in turn, a unique SE-specific set of *ARF*s might be triggered. To illustrate the assumptive differences in auxin responses in the early ZE and SE, we observed a distinct down-regulation of *ARF2* and *ARF3* during an early stage of SE that contrasted with the high expression of these genes in a zygotic cell of Arabidopsis (Rademacher et al. 2011; Xiang et al. 2011).

### SE-involved candidate *ARFs*—implications from the mutant analysis

To validate the SE-associated functions of the *ARF*s that had an SE-modulated expression pattern, the *arf* mutants were evaluated in terms of their embryogenic potential. The analysis showed that in spite of the extensive functional redundancy of the *ARF*s that were indicated in the developmental processes (Hardtke et al. 2004; Guilfoyle and Hagen 2007), the majority of the *arf* mutants were found to be defective in the SE response. These distinct SE-phenotypes of individual *arf* mutants suggest that the SE-related functions of the ARF proteins are not simply interchangeable as was also postulated in ZE (Rademacher et al. 2012).

Several *arf* mutants including *arf10* and *arf16* were found not to be affected in embryogenic potential, although a significant and auxin-dependent accumulation of *ARF10* and *ARF16* transcripts was associated with SE induction (present results). Thus, it may be possible that similar to *in planta* development (Wang et al. 2005) the *ARF10* and *ARF16* genes also act redundantly in SE. In support of an *ARF16* and *ARF10*, function in SE is the fact that these genes, together with *IAA17*/*AXR3*, act upstream of the *PLETHORA* (*PLT*) genes that regulate stem cell differentiation in Arabidopsis roots (Ding and Friml 2010) and have an assumed interaction with auxin in the control of the early SE (Horstman et al. 2014). Relevantly to this assumption, the significant accumulation of *ARF16* and *ARF10* transcripts (present result) was accompanied by an increased expression of the *IAA17*/*AXR3, PLT1*, and *PLT2* in an embryogenic culture of Arabidopsis (Gliwicka et al. 2013; Wickramasuriya and Dunwell 2015). Further support for the engagement of *ARF10* and *ARF16* in SE is that the up-regulation of these genes in the SE of Arabidopsis and other plants is accompanied by the down-regulation of miR160, which is a post-transcriptional regulator of *ARF10* and *ARF16* expression *in planta *(Mallory et al. 2005; Zhang et al. 2012; Lin and Lai 2013; Szyrajew et al. 2017).

The present results showed that mutations in *ARF1* and *ARF3* that had a distinctly down-regulated transcription during SE resulted in a significantly impaired embryogenic response. Similarly, a knockout mutation in the SE-engaged gene, *ERF022*, which had a highly repressed transcription during SE, caused a significantly reduced embryogenic response (Nowak et al. 2015). To interpret these unobvious effects of the SE-modulated genes with regulatory function, fine-tuning of the gene transcript level to that required for the promotion of the embryogenic transition might be considered. In support of this explanation, a specific level of *PLT* genes transcription was indicated to be associated with embryogenic response induced in culture of Arabidopsis explants (Horstman et al. 2015). Thus, SE-promoting effect of *ARF*s might be dependent on their expression level and relevantly to this assumption in flower development, a specific level of *ARF5* was found to be required for transcriptional regulation of the *LEAFY* gene (Yamaguchi et al. 2016).

Analysis of the overexpressor lines might be more informative than mutant analysis in the identification of the redundant gene functions (Prelich 2012). In accordance with this expectation, a phenotype of the *arf2* mutant, similar to ZE (Rademacher et al. 2011), was not affected in SE, while the overexpression of *ARF2* resulted in a significant reduction of the embryogenic response (present results). Consistent with the general function of *ARF2* that is annotated to the repression of cell divisions (Schruff et al. 2006), we indicated a distinct down-regulation of this gene transcripts during SE. Another line of evidence that supports *ARF2* involvement in the SE was provided by reports on the contribution of this gene to hormone-related responses including the repression of auxin signaling (Lim et al. 2010) and the integration of the auxin and brassinosteroid pathways (Vert et al. 2008).

### *ARF5*/*MONOPTEROS *contribution to SE

Consistent with the global SE-transcriptome analysis, we found *ARF5*, which encodes the MONOPTEROS (MP) protein, to be the most highly expressed member of the *ARF* gene family in the embryogenic culture of Arabidopsis. An increased activity level of *MP* was also reported in an embryogenic culture of soybean (Thibaud-Nissen et al. 2003). Thus, MP, similar to its fundamental role in the regulation of different aspects of ZE in Arabidopsis (Hardtke and Berleth 1998; Aida et al. 2002; Schlereth et al. 2010) seems to control the development of the somatic embryo. Evidence about a possible function of MP in auxin signaling during SE was provided recently by the indicated regulatory relation between MP and *PHABULOSA* (*PHB*), which is a positive regulator of *LEC2* that has an essential function in SE induction (Tang et al. 2012; Wójcikowska et al. 2013; Müller et al. 2015). In support of the regulatory impact of MP on the PHB-LEC2, pathway in SE is the up-regulation of *PHB* that was observed in an embryogenic culture of Arabidopsis (A. Wójcik, MDG, data not published) as well as the significantly reduced *LEC2* transcript level in an *mp*/*arf5* mutant (present results).

MP might also contribute to the embryogenic development that is triggered in plant somatic cells by controlling other *TF* genes that have a documented role during *in planta *development, including *HOMEOBOX GENE 8* (*ATHB8*) and *TARGET OF MONOPTEROS3* (*TMO3*), *TMO5, TMO6*, and *TMO7* (Donner et al. 2009; Schlereth et al. 2010). All of these MP targets were found to be up-regulated during SE in Arabidopsis (Gliwicka et al. 2013; Wickramasuriya and Dunwell 2015).

Similar to other ARFs, the developmental specificity of ARF5/MP function is generated by interactions with Aux/IAA proteins (Kieffer et al. 2010; Weijers et al. 2005), and many members of the Aux/IAA family were indicated to interact with MP in Arabidopsis (Krogan et al. 2014). Among the SE-involved AUX/IAA candidates is IAA30, which interacts with MP in ZE (Müller et al. 2015), and in support of its role in SE, a significantly impaired embryogenic response of the *iaa30* mutant was indicated (Gliwicka et al. 2013). Moreover, a regulatory relation between MP and BDL/IAA12 might be of importance for the SE induction mechanism as this protein pair is a major effector of auxin action in the zygotic embryo that influences the auxin-mediated cell-fate decisions in the early embryogenesis (Lau et al. 2011).

The function of MP in SE may also involve the regulation of the polar auxin transport and disturbed auxin transport was indicated to distinctly impair the embryogenic response of cultured explants (Chen and Chang 2004; Cueva-Agila et al. 2016). The MP-mediated control of the *PIN1* (*PIN-FORMED1*) gene that encoded the auxin efflux carrier was reported and a feedback regulatory loop that involves auxin, MP, and PIN1 was proposed (Wenzel et al. 2007; Krogan et al. 2016). Relevant to the assumed role of MP-mediated auxin transport pathway in SE, explants of a *pin1-7* mutant were indicated to be defective in embryogenic induction in vitro (Su et al. 2009). Moreover, the 35S::ARF5 line that resembles the *pin1* phenotypes (Hardtke et al. 2004) was found to be incapable of SE induction (present results). In conclusion, the strong inhibition of embryogenic potential that was observed in this study in the *mp*/*arf5* mutant and in the 35S::ARF5 line possibly result from the disturbed auxin signaling and impaired auxin transport that are expected in these forms (Mattsson et al. 2003; Lau et al. 2011).

Besides auxin, the possible contributions of MP in SE also involve the regulation of the cytokinin response pathway due to the involvement of MP in the regulation of cytokinin responses during plant development *in planta* and in vitro (Zhao et al. 2010; Ckurshumova and Berleth 2015). Considering that the patterning and cell organization that are associated with de novo regenerated shoot meristems resemble the formation of the embryonic SAM (Gordon et al. 2007), a cytokinin signaling-related function of MP in the development of the somatic embryo might also be expected. The distinctly up-regulated expression of *ARF10* in SE (this study), which positively regulates de novo shoot regeneration via the activation of the shoot meristem-specific genes, supports this assumption (Qiao et al. 2012).

In conclusion, ARF5/MP is assumed to control SE via versatile pathways (Fig. S6) and further analyses are needed to experimentally verify which of the MP-mediated regulatory interactions occur in an embryogenic culture.

## Conclusions

The study provides several pieces of evidence that numerous *ARFs*, which are the core regulators of auxin response, contribute significantly to the embryogenic switch that is induced in plant somatic cells in vitro. The candidate *ARF*s provide guidelines for further research on the auxin-mediated regulation of SE. Among the SE-involved candidates, the *ARF5* encoding MP protein that has a substantial role in the development of the zygotic embryo seems to significantly contribute to SE.

In the regulation of SE, similar to the *in planta* development including ZE, not only do the activities of individual ARFs need to be considered, but also the total ARF complement of a cell may create a pre-pattern that determines the SE-specific cellular response (reviewed in Chandler 2016). Therefore, one current challenge in deciphering the SE-related auxin response is the identification of the spatio-temporal and cell-specific sets of the ARFs and the interacting AUX/IAA elements that contribute to the embryogenic transition of somatic cells. To understand the biological functions of ARFs in SE, the ARF-targeted *TF* genes are required to be identified. In addition, the miRNAs and chromatin remodelers that control the SE-involved *ARF*s need to be uncovered considering the recently indicated key role of the post-transcriptional regulation of *ARF*s in plant development (Mallory et al. 2005; Oh et al. 2014).

### Author contribution statement

Małgorzata Danuta Gaj and Barbara Wójcikowska: (i) conceived and designed the experiments, and (ii) wrote the manuscript. Barbara Wójcikowska: performed the experiments and analyzed the data. All of the authors read and approved the manuscript.

## Electronic supplementary material

Below is the link to the electronic supplementary material.

**Table S1.** The insertional mutants, reporter, overexpressor lines, the primers used in PCR analysis and a checklist outlining the RNA to RT-qPCR quality/methodology. (xlsx 28 kb)

**Table S2.** Summarised results of the expression profile of* ARF* genes during SE process. (xlsx 11 kb)

**Table S3.** Analysis of the presence of AuxRE in the promoters of the SE-transcribed *ARF* genes. (DOCX 21 kb)

**Figure S1.** Qualitative RT-PCR analysis of ARF transcripts at different time points (0, 3, 5, 10, 15d) of the embryogenic Col-0 cultures induced on an E5 medium. RNA samples for the analysis were isolated from 0-, 3-, 5-, 10-, 15-day-old cultures. The *TIN *gene was used as the control for cDNA synthesis (n = 3). (TIFF 1024 kb)

**Figure S2.** Transcript levels of *ARF *genes during the SE process that were analyzed using the RNA samples isolated from 0-, 3-, 5-, 10-, 15-day-old cultures. The expression level was calculated using the 45-Ct method. *EF1α* and *TIN* genes were used as the control for cDNA synthesis. Means and SD for three biological replicates are shown. (TIFF 4061 kb)

**Figure S3.** Stable expression of *ARF7 *(**a**), *ARF9* (**b**) and *ARF19* (**c**) genes during the SE process. IZE explants of Col-0 were cultured on an E5 medium and the tissue was sampled on the 0, 3, 5, 10, 15d of the culture. Relative transcript level was normalized to the internal control (*TIN* gene) and calibrated to the 0d culture. * – expression level significantly different to that observed at 0d at P<0.05. Means and SD for three biological replicates are shown. (TIFF 3275 kb)

**Figure S4.** Qualitative RT-PCR analysis of *ARF* transcripts in Col-0 IZE explants cultured on CIM/SIM medium to induce shoot organogenesis (ORG). RNA samples for the analysis were isolated on the 0, 3, 5, 10, 15d of the culture. The *TIN* gene was used as the control for cDNA synthesis (n = 3). (TIFF 1047 kb)

**Figure S5.** Expression of *LEC2* during the SE process that was induced in the culture of the* arf5* mutant. IZE explants were cultured on an E5 medium and the tissue was sampled on the 0, 3, 5, 10, 15d. The relative transcript level was normalized to the internal control (*TIN* gene) and calibrated to the WT (Col-0) culture. * – expression level significantly different from that observed in the WT culture of the same age at P<0.05. Means and SD for three biological replicates are shown. (TIFF 4865 kb)

**Figure S6.** A model of the ARF5-controlled pathways that are possibly involved in SE induction. It is proposed that auxin-stimulated ARF5 regulates the expression of numerous genes that are involved in SE induction including *LEC2* (*LEAFY COTYLEDON2*) (presented results), which is an activator of the *YUC1*, *YUC4*, *YUC10 *(*YUCCA*) genes that are involved in auxin biosynthesis in SE (Wójcikowska et al. 2013) and* PIN1* (*PIN-FORMED1*), which is involved in polar auxin transport and positively controls SE (Su et al. 2009). In addition, the involvement of other ARF5-controlled TFs, including *ATHB8* (*HOMEOBOX GENE8*) and *TMO3*, *TMO5*, *TMO6*, *TMO7* (*TARGET OF MONOPTEROS*) (Schlereth et al. 2010) might also be considered in SE (Gliwicka et al. 2013). *Aux/IAA* genes (*IAA29*, *IAA30*, *IAA31*), which interact with ARF5 via the regulatory feedback loop (Krogan and Berleth 2015) have also been indicated as contributing to SE induction (Gliwicka et al. 2013). Solid line – experimentally confirmed interaction. Dashed line – interaction that needs to be confirmed.

## Abbreviations

2,4-D: 2,4-Dichlorophenoxyacetic acid
ARF: AUXIN RESPONSE FACTOR
Aux/IAA: AUXIN/INDOLE-3-ACETIC ACID
CIM: Callus induction medium
Ct: Threshold cycle
E0: Auxin-free induction medium
E5: Embryogenesis induction medium with auxin
IZE: Immature zygotic embryo
NAA: 1-Naphthaleneacetic acid
ORG: Organogenesis
PGR: Plant growth regulator
SAM: Shoot apical meristem
SE: Somatic embryogenesis
SIM: Shoot induction medium
TF: Transcription factor
ZE: Zygotic embryogenesis
