# Supplementary material for: Expression profiling of AUXIN RESPONSE FACTOR genes during somatic embryogenesis induction in Arabidopsis
Source: Plant Cell Rep. 2017 Mar 2;36(6):843–58. doi: 10.1007/s00299-017-2114-3 (PMC5486788; doi:10.1007/s00299-017-2114-3)
Supplement: Supplementary file 3 — Table S3. Analysis of the presence of AuxRE in the promoters of the SE-transcribed ARF genes. (DOCX 21 kb) [file 299_2017_2114_MOESM3_ESM.docx]

|  | ***ARF1*** | ***ARF2*** | ***ARF3*** | ***ARF5*** | ***ARF6*** | ***ARF7*** | ***ARF8*** | ***ARF9*** | ***ARF10*** | ***ARF11*** | ***ARF16*** | ***ARF17*** | ***ARF18*** | ***ARF19*** |
| --- | --- | --- | --- | --- | --- | --- | --- | --- | --- | --- | --- | --- | --- | --- |
| **Number of AuxRe elements in a gene promotor (AtcisDB)*** | 2 | 0 | 8 | 1 | 2 | 0 | 6 | 4 | 6 | 4 | 4 | 2 | 2 | 6 |
| **Experimentally validated *ARF* response to auxin** | - | - | - | Wenzel et al. 2007 | **-** | - | - | **-** | - | - | Paponov et al. 2008 | - | - | Paponov  et al. 2008  Chapman et al. 2012 |

* - http://arabidopsis.med.ohio-state.edu/AtcisDB/

Chapman EJ, Greenham K, Castillejo C, Sartor R, Bialy A, Sun TP, Estelle M (2012) Hypocotyl transcriptome reveals auxin regulation of growth-promoting genes through GA-dependent and-independent pathways. PLoS One 7:e36210

Paponov IA, Paponov M, Teale W, Menges M, Chakrabortee S, Murray JA, Palme K (2008) Comprehensive transcriptome analysis of auxin responses in Arabidopsis. Molecular Plant 1:321-337

Wenzel CL, Schuetz M, Yu Q, Mattsson J (2007) Dynamics of MONOPTEROS and PIN‐FORMED1 expression during leaf vein pattern formation in *Arabidopsis thaliana*. The Plant Journal 49:387-398
