# Supplementary figures and images for: Expression profiling of AUXIN RESPONSE FACTOR genes during somatic embryogenesis induction in Arabidopsis
Source: Plant Cell Rep. 2017 Mar 2;36(6):843–58. doi: 10.1007/s00299-017-2114-3 (PMC5486788; doi:10.1007/s00299-017-2114-3)

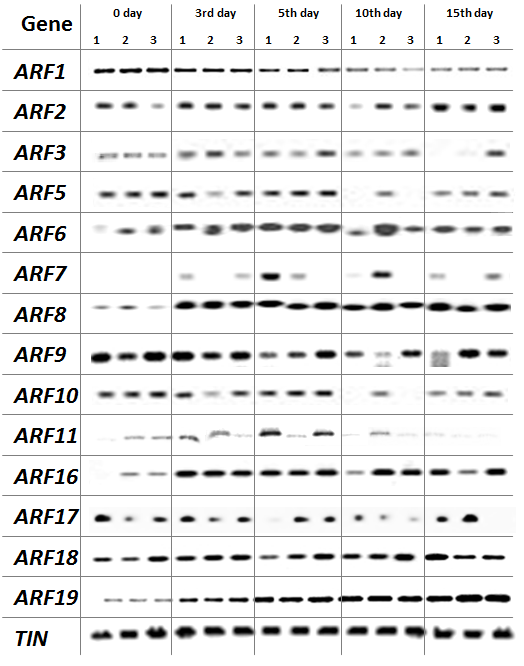

Supplement: Supplementary file 4 — Figure S1. Qualitative RT-PCR analysis of ARF transcripts at different time points (0, 3, 5, 10, 15d) of the embryogenic Col-0 cultures induced on an E5 medium. RNA samples for the analysis were isolated from 0-, 3-, 5-, 10-, 15-day-old cultures. The TIN gene was used as the control for cDNA synthesis (n = 3). (TIFF 1024 kb) [file 299_2017_2114_MOESM4_ESM.tif]

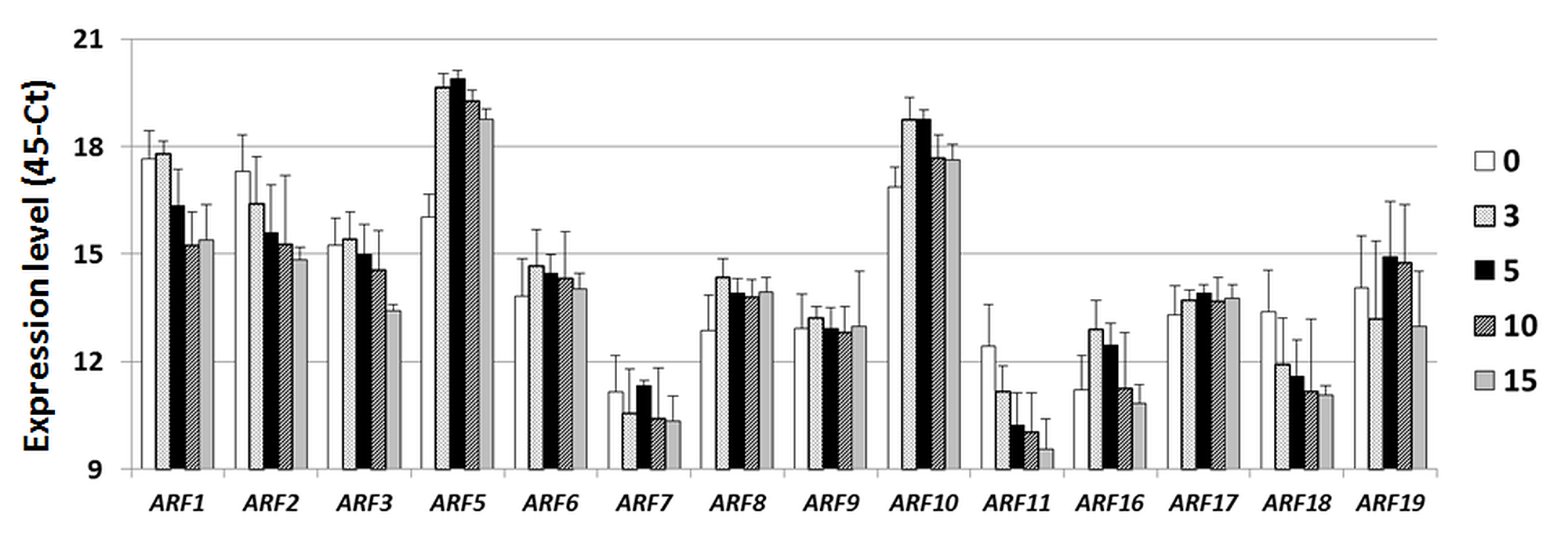

Supplement: Supplementary file 5 — Figure S2. Transcript levels of ARF genes during the SE process that were analyzed using the RNA samples isolated from 0-, 3-, 5-, 10-, 15-day-old cultures. The expression level was calculated using the 45-Ct method. EF1α and TIN genes were used as the control for cDNA synthesis. Means and SD for three biological replicates are shown. (TIFF 4061 kb) [file 299_2017_2114_MOESM5_ESM.tif]

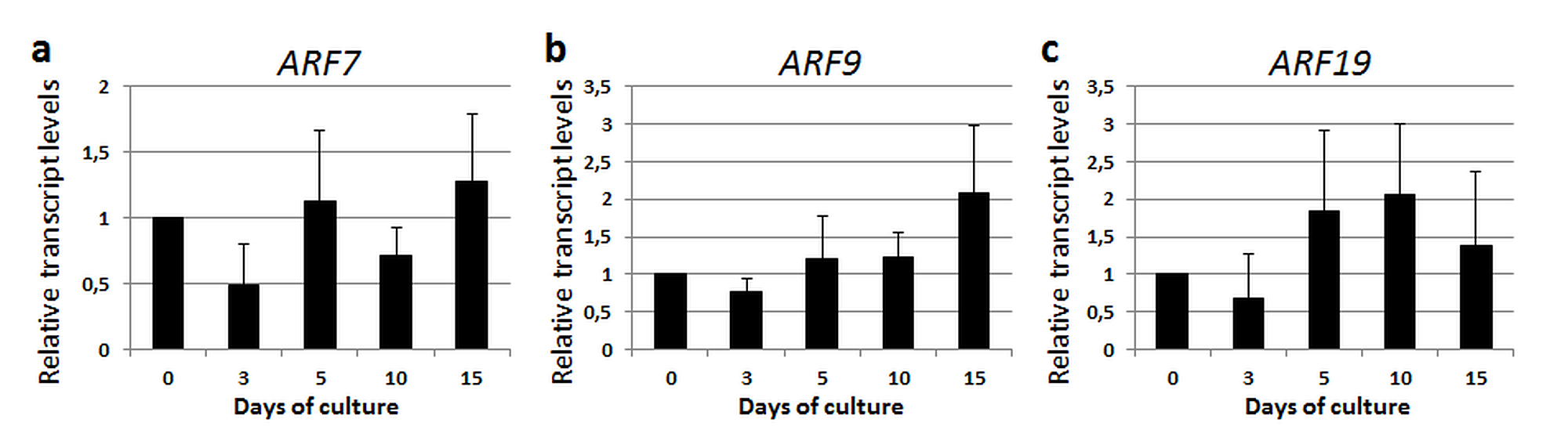

Supplement: Supplementary file 6 — Figure S3. Stable expression of ARF7 (a), ARF9 (b) and ARF19 (c) genes during the SE process. IZE explants of Col-0 were cultured on an E5 medium and the tissue was sampled on the 0, 3, 5, 10, 15d of the culture. Relative transcript level was normalized to the internal control (TIN gene) and calibrated to the 0d culture. * – expression level significantly different to that observed at 0d at P<0.05. Means and SD for three biological replicates are shown. (TIFF 3275 kb) [file 299_2017_2114_MOESM6_ESM.tif]

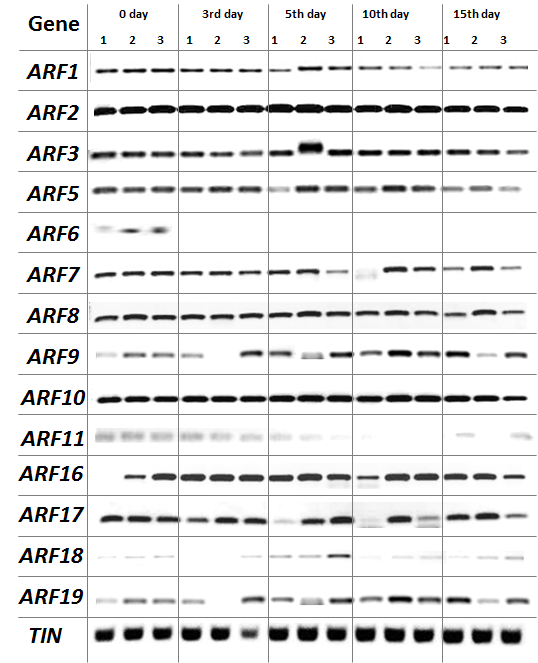

Supplement: Supplementary file 7 — Figure S4. Qualitative RT-PCR analysis of ARF transcripts in Col-0 IZE explants cultured on CIM/SIM medium to induce shoot organogenesis (ORG). RNA samples for the analysis were isolated on the 0, 3, 5, 10, 15d of the culture. The TIN gene was used as the control for cDNA synthesis (n = 3). (TIFF 1047 kb) [file 299_2017_2114_MOESM7_ESM.tif]

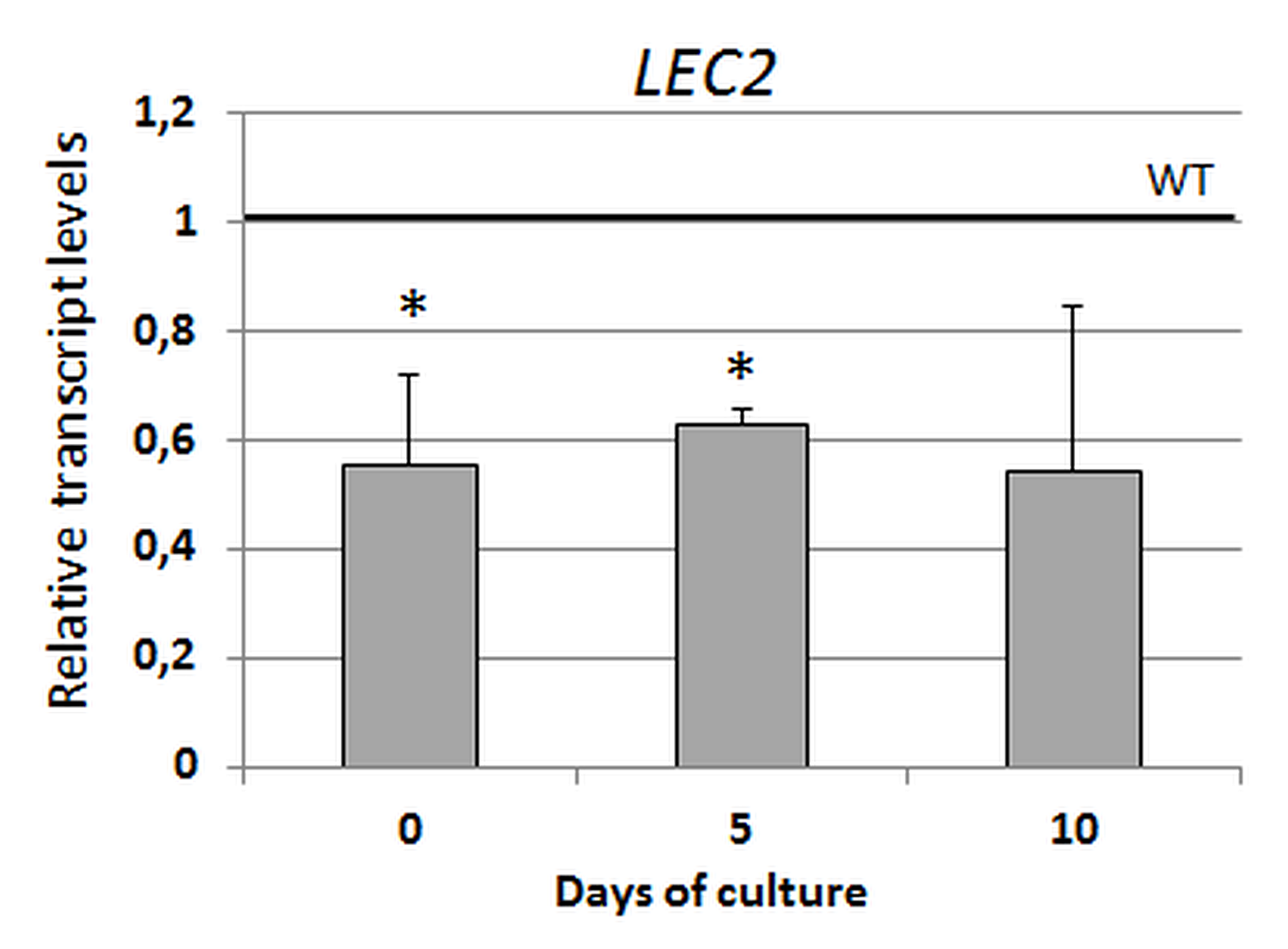

Supplement: Supplementary file 8 — Figure S5. Expression of LEC2 during the SE process that was induced in the culture of the arf5 mutant. IZE explants were cultured on an E5 medium and the tissue was sampled on the 0, 3, 5, 10, 15d. The relative transcript level was normalized to the internal control (TIN gene) and calibrated to the WT (Col-0) culture. * – expression level significantly different from that observed in the WT culture of the same age at P<0.05. Means and SD for three biological replicates are shown. (TIFF 4865 kb) [file 299_2017_2114_MOESM8_ESM.tif]

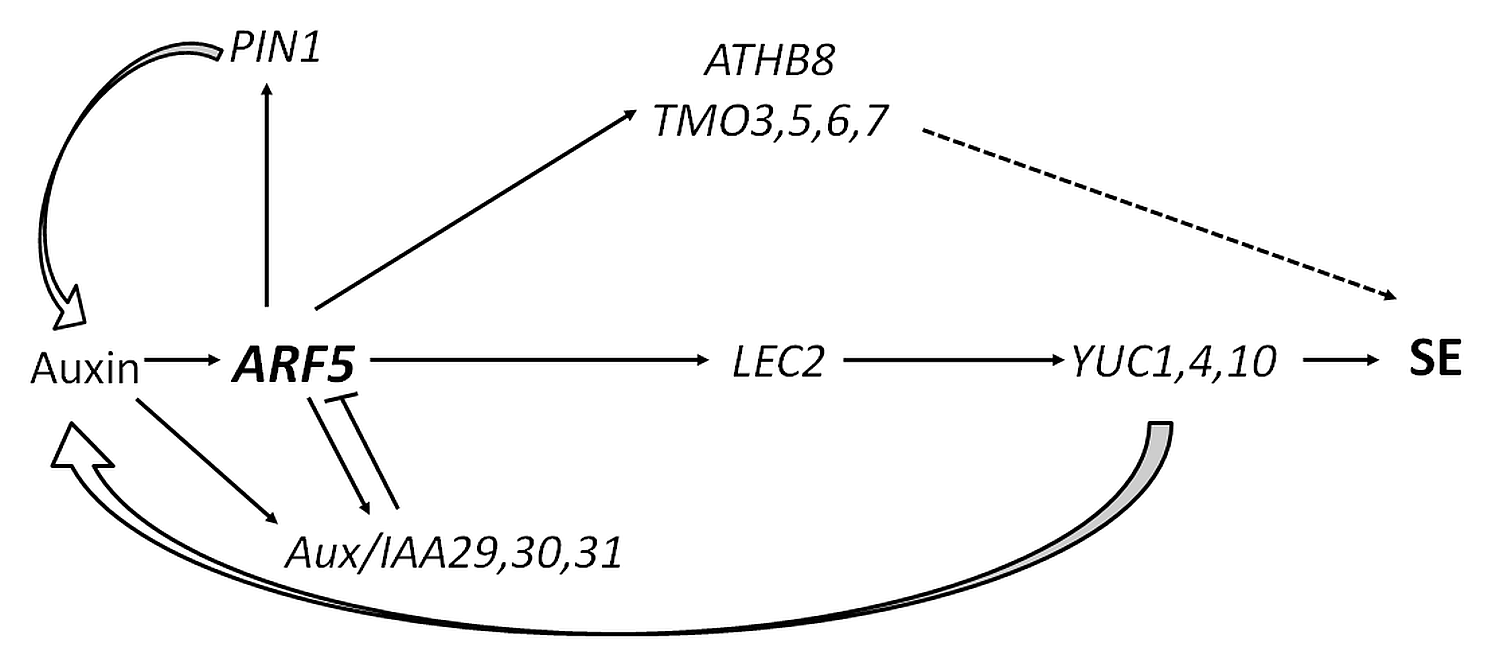

Supplement: Supplementary file 9 — Figure S6. A model of the ARF5-controlled pathways that are possibly involved in SE induction. It is proposed that auxin-stimulated ARF5 regulates the expression of numerous genes that are involved in SE induction including LEC2 (LEAFY COTYLEDON2) (presented results), which is an activator of the YUC1, YUC4, YUC10 (YUCCA) genes that are involved in auxin biosynthesis in SE (Wójcikowska et al. 2013) and PIN1 (PIN-FORMED1), which is involved in polar auxin transport and positively controls SE (Su et al. 2009). In addition, the involvement of other ARF5-controlled TFs, including ATHB8 (HOMEOBOX GENE8) and TMO3, TMO5, TMO6, TMO7 (TARGET OF MONOPTEROS) (Schlereth et al. 2010) might also be considered in SE (Gliwicka et al. 2013). Aux/IAA genes (IAA29, IAA30, IAA31), which interact with ARF5 via the regulatory feedback loop (Krogan and Berleth 2015) have also been indicated as contributing to SE induction (Gliwicka et al. 2013). Solid line – experimentally confirmed interaction. Dashed line – interaction that needs to be confirmed. [file 299_2017_2114_MOESM9_ESM.tif]
